# Grand Challenge: How Do We Dodge the Obesity Armageddon?

**DOI:** 10.3389/fendo.2016.00119

**Published:** 2016-08-24

**Authors:** Katherine Samaras

**Affiliations:** ^1^Diabetes and Obesity Program, Garvan Institute of Medical Research, Darlinghurst, NSW, Australia; ^2^Department of Endocrinology, St Vincent’s Hospital, Darlinghurst, NSW, Australia

**Keywords:** obesity, endocrinology, public health, epidemiology, nutrition

“To raise new questions, new possibilities, to regard old problems from a new angle, requires creative imagination and marks real advance in science.”

Albert Einstein

To find new questions and new possibilities in the field of obesity, that is the Grand Challenge, the gauntlet I humbly place before us, researchers examining the genetic, epigenetic, molecular, cellular, physiological, psychological, nutritional, pharmacologic, environmental, electronic, economic, population, geographic, or epidemiologic aspects of this pandemic, clinicians at the coal face treating the myriad of diseases either caused by or accelerated by obesity, industry producing foods or products to ameliorate the crisis (rather than exacerbate it) or policy makers and politicians charged with steering our local, national, and international ship to safer waters. The solution to our crisis in obesity and its fall outs requires these and many other players to build on the evidence that informs innovative scientific developments, novel treatments, and prevention approaches and guides health policy and political commitment.

Obesity today threatens to undermine the benefits of two centuries of improved longevity and human health won by hygiene, water safety, nutrition, antibiosis, immunization, smoking cessation, and medical care. Overweight and obesity have reached epidemic proportions worldwide, affecting 39% of adults, an estimated 1.9 billion people of whom 600 million are obese (1). In the United States of America, rates of adult overweight and obesity exceed 68% (2). The most recent reports in a sobering corpus of evidence show that obesity afflicts 38% of adults (3) and 17% of children and adolescents (4). Of concern, there is evidence that obesity prevalence continues to grow in women and adolescents (3, 4). Obesity prevalence rates in low- and middle-income countries have not been spared the upsurge in obesity prevalence.

*Now imagine if all that could change*. Imagine if we could alter the energy balance equation at any of the multitude of opportunities that exist from agriculture to food production, to the consumer as a mass and as individuals, to the mouth, the brain, the gut, the cell, the mitochondrium, the DNA, and how it is decoded, read, amplified, or silenced; how energy is utilized, expended, and how an excess might be stored or perhaps not stored. How the effects of excess energy on health and disease might be blunted. How the health of those affected can be ameliorated, improved, or cured; how the unaffected can remain so. These are the challenges that are ahead that require vision, effort, enterprise, commitment, and endurance across a united front of multiple disciplines.

*Frontiers in Endocrinology, Obesity* will be an inclusive vehicle for researchers from the multiple and wide disciplines relevant to obesity, to address the new and old questions, with curiosity, imagination (the forethought to innovation), and rigor in scientific excellence, even in these times of research funding insecurity. A vision forward, to improved knowledge, understanding, and practice.

The Grand Challenge is to champion innovative science in obesity, to integrate scientific effort between the disciplines, to promote translation of basic science to the best standard of clinical practice in serving our patients, to inform policy makers and political leaders and advocate for our obese patients who are the raison d’etre for this mandate. Our challenge is to champion the health and outcomes of the obese and to contribute to the solutions to the health and social crisis we find ourselves in.

## Author Contributions

The author confirms being the sole contributor of this work and approved it for publication.

## Conflict of Interest Statement

The author declares that the research was conducted in the absence of any commercial or financial relationships that could be construed as a potential conflict of interest.

## References

[1] World Health Organisation. Obesity and Overweight Fact Sheet (Updated June 2016). (2016). Available from: http://www.who.int/mediacentre/factsheets/fs311/en/

[2] Flegal KM Carroll MD Kit BK Ogden CL. Prevalence of obesity and trends in the distribution of body mass index among US adults, 1999-2010. JAMA (2012) 307(5):491–7. 10.1001/jama.2012.39 22253363

[3] Flegal KM Kruszon-Moran D Carroll MD Fryar CD Ogden CL. Trends in obesity among adults in the United States, 2005 to 2014. JAMA (2016) 315(21):2284–91. 10.1001/jama.2016.6458 27272580 PMC11197437

[4] Ogden CL Carroll MD Lawman HG Fryar CD Kruszon-Moran D Kit BK Trends in obesity prevalence among children and adolescents in the United States, 1988-1994 through 2013-2014. JAMA (2016) 315(21):2292–9. 10.1001/jama.2016.6361 27272581 PMC6361521

